# Open questions on carbonaceous matter in meteorites

**DOI:** 10.1038/s42004-024-01200-8

**Published:** 2024-05-29

**Authors:** Oliver Christ, Fabrizio Nestola, Matteo Alvaro

**Affiliations:** 1 Department of Earth and Environmental Sciences, University of Pavia, 27100 Pavia, Italy; 2 Department of Geosciences, University of Padua, 35131 Padua, Italy

**Keywords:** Origin of life, Solid-state chemistry, Astrochemistry

## Abstract

Extraterrestrial carbon gives insights into the origin of life and processes that took place billions of years ago in our solar system. Here, the authors provide an overview of what is known and of unanswered questions with a meteoritical focus.

## Origin and occurrence of carbon in meteorites

**Fig. 1:**
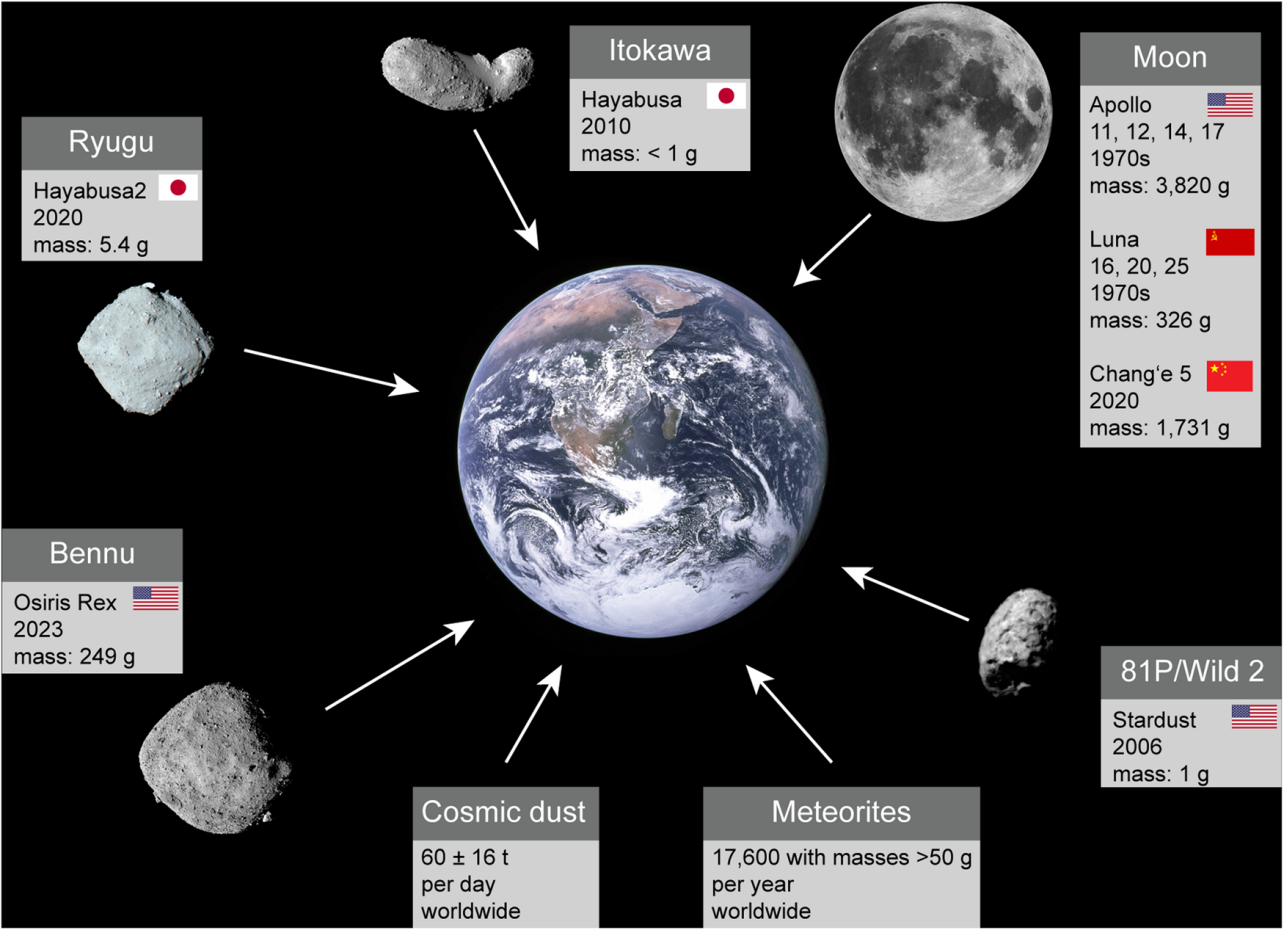
Influx of extraterrestrial material to Earth. Influx of extraterrestrial material to Earth, including all successfully conducted sample return missions since the Apollo 11 mission in 1969. Of these, only the Apollo missions by NASA were manned, hence the comparably high mass of returned material. Masses for the Apollo and Luna missions are summed up. Celestial bodies are not to scale. Image credits: Bennu: NASA/Goddard/University of Arizona; Earth, Moon: NASA; 81P/Wild 2: NASA/JPL-Caltech; Itokawa, Ryugu: ISAS/JAXA.

Understanding our solar system has been a driving force for different fields of planetary science, such as astrophysics, astrobiology, and planetary geoscience. While astrophysicists mainly work with electromagnetic radiation of different wavelengths and frequencies, astrobiologists and planetary geoscientists can work directly with extraterrestrial materials. These materials can be brought to Earth either through sample return missions such as the manned Apollo missions to the Moon by NASA in the 1970s or sample return missions with unmanned spacecrafts to the Moon (Luna 16, 18, and 20 by the Soviet Union in the 1970s), comets (Stardust by NASA), or asteroids (Hayabusa and Hayabusa2 by JAXA or OSIRIS-Rex by NASA). However, the amount of material brought back by robotic missions is rather small (Fig. 1), but fortunately, about 60 t of cosmic/interplanetary dust particles (IDP) with sizes of 1–50 µm^1^ land on Earth’s surface per day^2^ followed by ~17,600 meteorites with a mass of >50 g per year^3^ and micrometeorites (sizes <1000 µm^4^). (Micro-)Meteorites and IDPs have long been used to answer questions regarding the formation of our solar system and the origin of life. For these purposes carbon bearing phases such as amino acids and inorganic carbon polymorphs have played a major role. However, despite numerous studies on extraterrestrial carbon there are still open questions for both carbon-based molecules in space^5^ and in meteorites.

Carbon is the fourth most abundant element in the solar photosphere and the fifth most abundant in meteorites^6^. In these stones of extraterrestrial origin, carbon can be found in both major groups of undifferentiated chondrites and differentiated achondrites and in the latter also in both subgroups of stony and iron meteorites^7^. Carbon, like all elements heavier than ^7^Li, is generated in stars through either stellar evolution (up to ^56^Fe) or stellar explosions (elements beyond ^56^Fe)^8^. The production of ^12^C involves two steps during the He-burning phase within a red giant^9^. In the initial step, two alpha particles combine to form ^8^Be, followed by the capture of an additional alpha particle, leading to the disintegration into ^12^C^10^.

Since this process requires a red giant, the elements in our solar system were not produced by the Sun but by a red giant that eventually died as a supernova. The supernova generated an interstellar molecular cloud containing gas and dust. Subsequently, this molecular cloud collapsed, resulting in the formation of the proto-Sun accumulating 99% of the mass and a protoplanetary disk surrounding it. Within the protoplanetary disk, dust grains began to aggregate, forming centimetre-sized objects that eventually grew into objects of up to 100 km in size. Planetesimals formed, which then accreted into planetary embryos that eventually developed into the planets present today. The accretion of sufficient material eventually led to differentiation, i.e., the formation of a core, a mantle, and a crust, as we know it from our planet Earth. Responsible for this process is mainly the radioactive decay of ^26^Al^11^ that produces temperatures that are high enough to melt the constituents of primitive celestial bodies and thus overprint pristine features, including carbon. However, not all celestial bodies underwent differentiation, as we can see for asteroids in the asteroid belt between Mars and Jupiter, which were simply too small. Here, asteroids—the parent bodies of chondrites—still contain unprocessed material from the early stages of our solar system in the form of organic compounds and dust grains. These rare grains, known as pre-solar grains, occur in the form of diamond, graphite and SiC in carbonaceous^12^ and primitive^13^ chondrites and are characterised by unusual isotopic noble gas signatures which do not align with the bulk composition of the Sun^14^.

Due to continuous impact events in our solar system, material from the impacted celestial body gets ejected and can end up on Earth’s surface in the form of meteorites. Nowadays, more than 70,000 different meteorites, of which, for some, the parent body could be identified to be Moon, Mars, the asteroid 4 Vesta, or specific asteroid types, are listed in Earth’s meteorite collection. Here, carbon occurs as both native (i.e., graphite and diamond) and bound within minerals like carbonates (e.g., calcite CaCO_3_, siderite FeCO_3_) and carbides (e.g., cohenite (Fe,Ni,Co)_3_C, haxonite (Ni,Fe)_23_C_6_, SiC). In addition, two more names are worth mentioning: cliftonite and lonsdaleite^15^. While cliftonite describes the phenomenon when graphite pseudomorphs kamacite in iron meteorites, lonsdaleite, often referred to as a hexagonal diamond, is produced when graphite is subjected to high pressure and transforms into diamond. However, there is considerable doubt if this phase exists as a discrete mineral^15^. Besides its native and mineral occurrence, carbon can be found in a variety of organic compounds in carbonaceous chondrites^16–18^ and achondrites^19^, as mentioned earlier.

## Carbon and the origin of life

Organic compounds of extraterrestrial origin are known from asteroids, comets, meteorites, and IDPs and have long been assumed to represent the first step toward terrestrial life. The initial delivery of such organic material to Earth most likely took place during the late heavy bombardment^20^ when the inner solar system was bombarded by asteroids and comets 3.5–4.2 Ga ago, with terrestrial impacts lasting until up to 2.0 Ga ago. Apart from meteorites, IDPs, which can be collected in Earth’s stratosphere^21^, are an important carrier for organic compounds toward Earth as they contain large percentages of carbon, including organic compounds^22^.

The first report of organic compounds in a rock of extraterrestrial origin dates back to 1806 in the Alais meteorite^23^, the very first carbonaceous chondrite ever found. However, the first report of amino acids of undoubtable extraterrestrial origin emerged after the observed fall (which limits terrestrial weathering compared to simple meteorite funds) in 1969 of the carbonaceous CM2 chondrite Murchison, of which 100 kg were recovered in Australia^24^. In carbonaceous chondrites, carbon accounts for 1.5-6 wt%^25,26^, where organic compounds, including amino acids, are the primary carrier of carbonaceous matter.

A crucial point in establishing a link between extraterrestrial organic matter and terrestrial life was the chirality, i.e., the occurrence of the left (l) and right (d) handed forms of amino acids and the fact that amino acids in meteorites show an excess of l-amino acids^27–29^. Abiotic processes lead towards a 50/50 ratio of l- and d-amino acids, also called racemic ratio, while terrestrial biological life produces preferably l-amino acids, just like amino acids found in meteorites. Organic compounds in carbonaceous chondrites, however, are not limited to just amino acids. Moreover, they include a variety of different classes such as carboxylic acids, hydroxy acids, sugar-related, phosphorus, and sulfuric compounds, and many others^16,17,30–32^. Eventually, the discovery of these possible building blocks of terrestrial life paved the way for the hypothesis that organic matter transported by meteorites to Earth led to the formation of terrestrial life^33,34^.

In-situ space missions like Rosetta (comet 67P/Churyumov–Gerasimenko) and sample return missions like Stardust (comet 81P/Wild-2), Hayabusa2 (asteroid Ryugu), and OSIRIS-REx (asteroid Bennu) provided and will provide insights in the organic inventory of comets^35–37^ and asteroids^38,39^. Extraterrestrial organic matter is well studied and a variety of compounds has been reported. The exact link between these building blocks and actual terrestrial life, however, remains enigmatic^40^. The same applies to possible (past) extraterrestrial life, e.g., on Mars. Organic compounds from Mars are known from both Martian meteorites^41–43^ and the Martian surface from in-situ analyses by rovers^44–48^, a recent review about organic matter on Mars can be found in^49^ highlighting the importance of such analyses. A promising approach towards Martian life are ongoing (Mars 2020 with its Perseverance rover and the Mars Sample Return mission) and future space missions with the goal to not only further analyse in-situ but also to return Martian material (e.g., China’s Tianwen-3 mission). In addition to Martian rocks and soil, the exploration of Martian ice deposits is a high priority for future planetary missions, as ice cores from Mars hold the potential to offer insights into the planet’s past climate, geology, and astrobiology, and thus, life beyond Earth. However, the technology is not yet ready for ice core sample return missions.

## Carbon and the early solar system

### Stellar nucleosynthesis as seen by carbon

Apart from the origin of life, extraterrestrial carbon also helps to understand the processes of the early solar system as well as processes that took place long before its formation. For instance, pre-solar grains, with their unusual isotopic signatures, can be used to understand stellar evolution, nucleosynthesis, mixing in supernovae, galactic evolution, and the age of the galaxy^50^. For example, SiC grains contain information about H- and He-burning processes in the core and shells of red giant and asymptotic giant branch stars, which are long lost^50^. Pre-solar diamond and graphite, on the other hand, encapsulated isotopic signatures of supernovas in the form of Xe, ^44^Ti, and ^28^Si isotopes^50^. However, such grains are rare. Concentrations have been reported to be as low as 4530 ppm for SiC + spinel in the Axtell CV3 meteorite and 613 ppm for diamond in the Murchison CM2 meteorite^14^.

### Planetary formation and what carbon in meteorites can tell us about parent bodies

Further, carbon in meteorites also allows the unraveling of a variety of planetary formation processes^51^. Therefore, especially the isotopic distribution of carbon is of interest, which helps to identify primary, secondary, and tertiary carbon species. Primary carbon contains isotope ratios of the original source reservoirs, i.e., the molecular cloud, and occurs if they survived hydrous or thermal alteration in the form of organic molecules, amorphous/graphitic carbon, nanodiamonds, and SiC^51^. Secondary carbon species did not survive alteration processes and were, therefore, formed on the specific meteorite’s parent body. Isotopic signatures are, of course, linked to their precursors, i.e., primary species, and distinctions may be difficult^51^. Lastly, tertiary species are the product of the interaction of cosmic radiation and the parent body. A summary of δ^13^C values from different meteorite groups can be found in Grady and Wright (2003)^51^.

**Fig. 2:**
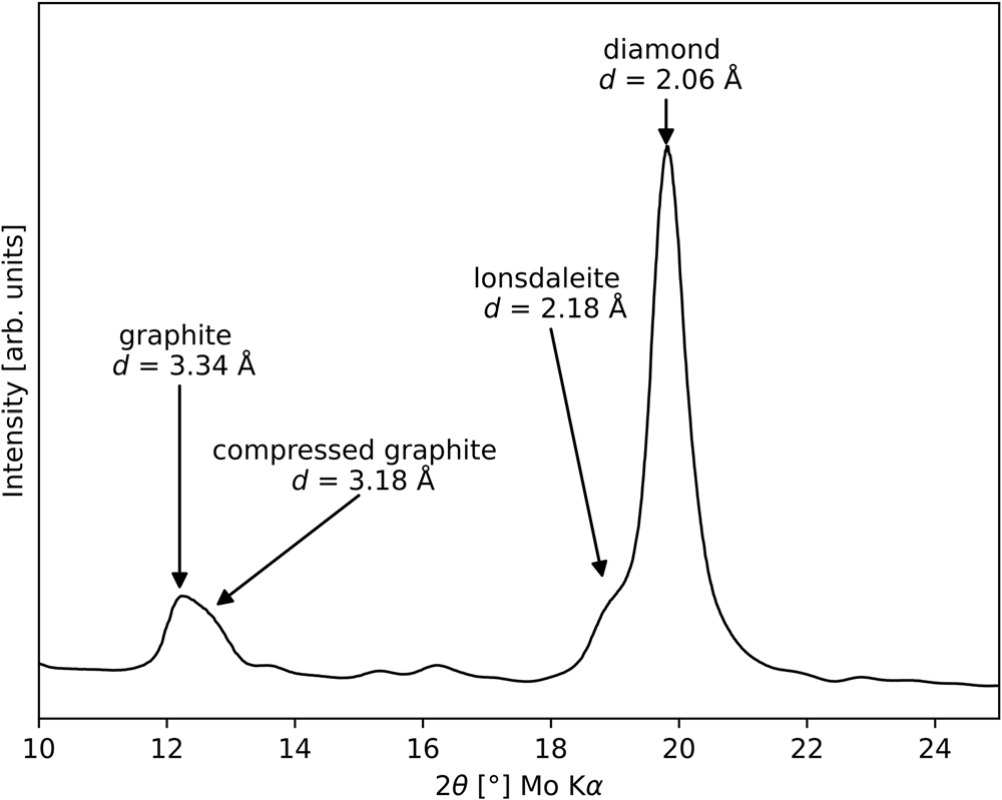
Exemplary diffractogram of a carbon-bearing aggregate. Diffractogram of a carbon-bearing aggregate from a highly shocked ureilite showing the two crystallographic shock indicators lonsdaleite and compressed graphite.

Among carbon-bearing achondrites, ureilites are of great interest as carbon shows significantly high abundances of up to 8 wt%^52^. Within ureilites, carbon-bearing aggregates are commonly found interstitial containing both graphite and diamond^53^. Only in unshocked ureilites graphite occurs solely without accompanying diamond^54^. Since the discovery of ureilitic diamonds in 1888^55^, these diamonds remain enigmatic. Many studies have been conducted on them to understand the evolution of the ureilite parent body (UPB) including its size of which estimations range from 150^56^ to 6779 km^57^ in diameter. Further discrepancies can be seen in the origin of the ureilitic diamond itself. Three distinct hypotheses for its formation process were postulated: (i) the formation deep inside a planetary body, analogue to terrestrial diamonds^58^, (ii) the direct formation from graphite during a shock event^59^, (iii) the formation by crystal vapour deposition^60^. Although option (ii) is the most accepted hypothesis, a consensus has not been reached yet, as recent publications show^53,57,61^. A new approach toward the diamond formation scenario was the application of graphite geothermometry. Initially developed for chondritic parent bodies^62^, graphite geothermometry has then been applied also to graphite in ureilites^63^. Following the first application on the famous Almahata Sitta ureilite originating from the 2008 TC3 asteroid, the first asteroid that has been completely observed from its identification in space to its impact in Sudan^64^, this method has been applied to different ureilites yielding temperatures between 1180 and 1398 °C^65–69^. These temperatures fall into the catalytic diamond synthesis field, as can be seen in the carbon phase diagram^70^, suggesting that with the help of Fe-bearing phases, which are widely available as they always accompany carbon-bearing aggregates in ureilites, this could be the temperature reached during the impact event which destroyed the UPB and formed ureilitic diamond directly from graphite. Further proof for this scenario can be seen in the presence of the shock indicators lonsdaleite^15^ and compressed graphite^71^. Both shock indicators can be seen in Fig. 2. Here, lonsdaleite appears as a shoulder of the most intense diamond reflection at *d*-spacing of 2.06 Å towards higher *d*-spacings in the range between 2.15 and 2.18 Å while compressed graphite shows a strong peak asymmetry of the most intense graphite reflection of 3.34 Å towards lower *d*-spacings around 3.18 Å.

Carbon isotopes from ureilites have also been analysed. However, diamond always occurs together with graphite, only graphite may occur solely. This renders the acquisition of δ^13^C values of pure diamond rather difficult. In the literature, only three reports of such values can be found, of which one is relatively old and one is a conference abstract^72–74^. Apart from these reports, the stepped combustion method has often been used to determine δ^13^C of both graphite and diamond. This method, however, faces the problem of overlapping combustion temperatures of the two carbon polymorphs^75^. In summary, δ^13^C values have been reported and range between −11.1‰ and +7.01‰ with two major peaks and have since then been used to constrain the UPB and to explain the origin of carbon in ureilites^51,72–81^.

The main carbon bearing phases in iron meteorites are carbides and graphite, only in three iron meteorites diamond has been reported^82–84^. Diamonds in iron meteorites have been interpreted as the result of impacts; in the case of Canyon Diablo, the iron meteorite that created the Barringer crater in Arizona, USA, these diamonds were formed when the meteorite hit Earth’s surface^84^. For the other two iron meteorites, ALHA77283 and Chuckwalla, diamonds were interpreted as pre-terrestrial impact diamonds^82,83^, i.e., impacts in space on the parent body produced these diamonds.

The discovery of diamond in Canyon Diablo also led to the discovery of hexagonal diamond or lonsdaleite^85^. Recently, the existence of lonsdaleite as a discrete mineral has been questioned and it was reinterpreted as faulted and nano twinned diamond^15^. The extent of such stacking faults can also be quantified^86^. Whether it is a distinct mineral or not, lonsdaleite has been found in numerous impact craters and has become a widely accepted impact/shock indicator^87–89^.

## Open challenges regarding extraterrestrial carbonaceous matter

Despite the various studies of extraterrestrial carbonaceous matter from different fields covering a variety of different aspects, many remain enigmatic. As previously mentioned, the origin of terrestrial life and the possibility of (past) Martian life remain open questions, even with all technological advances. Regarding high pressure minerals, extraterrestrial carbonaceous matter is limited to diamond, which mainly occurs in ureilites and chondrites. For ureilitic diamond, their origin is still a matter of debate and albeit iron meteorites contain graphite and must have experienced violent impact events or otherwise these metallic cores would not have been separated from their respective parent body, diamond is widely absent as it occurs in just three iron meteorites. The characterisation of such impact events is also rather difficult. At this point, pressures can be derived mainly from silicates, a mineral group that is, at best, an accessory in iron meteorites, and thus, a comparable pressure/shock classification scheme for all iron meteorite groups still does not exist. Regarding shock temperatures, only one geothermometer might exist so far, which, however, has not been calibrated for such conditions and has a relatively high uncertainty of ±120 °C. The calibration of this graphite-based geothermometer with shock pressures would be a promising approach.

The above mentioned absence of diamonds in iron meteorites needs to be understood as well. Here, graphite is a relatively common accessory mineral. However, it is widely unaltered, although iron meteorites must have experienced violent impact events. A possible explanation could be the different mechanical properties of the matrix constituting minerals kamacite and taenite with respect to forsterite (∼Mg_2_SiO_4_) in ureilites.

From a geochemical point of view, δ^13^C values of pure diamonds are needed as such data could help to shed light on their formation process. Although the preparation of clean diamond, i.e., without accompanying graphite, is not difficult, this process requires grains of significant size and unfortunately, extraterrestrial microdiamonds are extremely rare. However, promising samples can be found in the Meteoritical Bulletin, namely ureilites, which are reported to be hard to almost impossible to cut.

Whether addressing fundamental questions in astrobiology about the origin of terrestrial life and the potential existence of life beyond Earth or in planetary geoscience to comprehend the early stages of our solar system, extraterrestrial carbon remains a subject of significant interest. However, samples are often rare and preparation methods are often of a destructive nature. More material is clearly needed, preferably as uncontaminated by Earth’s atmosphere and surface as possible. The annual and nowadays well-monitored influx of meteorites could contribute to it, but often meteorites fall into the Earth’s oceans or, despite the monitoring systems, unnoticed and thus will be subject to terrestrial weathering until found. Future sample return missions, of course, are the solution of choice but also the most expensive one, and the yield of returned material is often small. At the time of writing, future sample return missions include the Chinese Chang’ 6, Tianwen-2, and Tianwen-3 missions to the Moon, the asteroid 469219 Kamoʻoalewa, and Mars, respectively, the Japanese MMX mission to the Martian moon Phobos, India’s Chandrayaan-4 mission to Moon and a sample return mission to Mars by NASA and ESA. Apart from these already planned and partly ongoing missions, NASA is planning to collect and return ice cores from Mars, which would be of major interest, especially for astrobiology, as microorganisms can be found in terrestrial ice cores.

The scientific and financial investments of space agencies all around the globe underline the importance of extraterrestrial material for our understanding of the solar system, and even though not all the above-mentioned sample return missions might have the goal of answering carbon-related questions, these samples could play important roles in our understanding of some aspects which involve extraterrestrial carbon and more broadly the origin of life.
